# Unattended compared to traditional blood pressure measurement in patients with rheumatoid arthritis: a randomised cross-over study

**DOI:** 10.1080/07853890.2021.1999493

**Published:** 2021-11-09

**Authors:** Elena Bartoloni, Fabio Angeli, Elisa Marcucci, Carlo Perricone, Giacomo Cafaro, Clara Riccini, Lorenzo Spighi, Benedetta Gildoni, Claudio Cavallini, Paolo Verdecchia, Roberto Gerli

**Affiliations:** a Rheumatology Unit, Department of Medicine and Surgery, University of Perugia, Perugia, Italy; b Department of Medicine and Surgery, University of Insubria and Department of Medicine and Cardiopulmonary Rehabilitation, Maugeri Care and Research Institutes, IRCCS Tradate, Varese, Italy; c Department of Cardiology, Hospital S. Maria della Misericordia, Perugia, Italy; d Fondazione Umbra Cuore e Ipertensione, Perugia, Italy

**Keywords:** Rheumatoid arthritis, hypertension, blood pressure measurement, left ventricular hypertrophy, electrocardiography, echocardiography

## Abstract

**Background:**

Hypertension is characterised by a high prevalence, low awareness and poor control among rheumatoid arthritis (RA) patients. Correct blood pressure (BP) measurement is highly important in these subjects. The “unattended” BP measurement aims to reduce the “white-coat effect,” a phenomenon associated with cardiovascular risk. Data on “unattended” BP measurement in RA and its impact on hypertensive organ damage are very limited.

**Methods:**

BP was measured in the same patient both traditionally (“attended” BP) and by the “unattended” protocol (3 automated office BP measurements, at 1-min intervals, after 5 min of rest, with patient left alone) by a randomised cross-over design. Patients underwent clinical examination, 12-lead electrocardiography and trans-thoracic echocardiography to evaluate cardiac damage.

**Results:**

Sixty-two RA patients (mean age 67 ± 9 years, 87% women) were enrolled. Hypertension was diagnosed in 79% and 66% of patients according to ACC/AHA and ESC/ESH criteria, respectively. Concordance correlation coefficients between the two techniques were 0.55 (95%, CI 0.38–0.68) for systolic BP and 0.73 (95%, CI 0.60–0.82) for diastolic BP. “Unattended” (121.7/68.6 mmHg) was lower than “attended” BP (130.5/72.8 mmHg) for systolic and diastolic BP (both *p* < .0001). Among the two techniques, only “unattended” systolic BP showed a significant association with left ventricular mass (*r* = 0.11; *p* = .40 for “attended” BP; *r* = 0.27; *p* = .036 for unattended BP; difference between slopes: *z* = 3.92; *p* = .0001).

**Conclusions:**

In RA patients, “unattended” BP is lower than traditional (“attended”) BP and more closely associated with LV mass. In these patients, the “unattended” automated BP measurement is a promising tool which requires further evaluation.

**KEY MESSAGES**
- “Unattended” automated blood pressure registration, aimed to reduce the “white-coat effect” is lower than “attended” value in rheumatoid arthritis patients.
- “Unattended” blood pressure is more closely associated with left ventricular mass than “attende” registration.

## Introduction

Several studies suggest that the risk of major cardiovascular (CV) events, such as stroke, myocardial infarction and congestive heart failure, is increased in patients with rheumatoid arthritis (RA) when compared with the general population [1]. Among the traditional CV risk factors, hypertension (HT) is a major and often underdiagnosed contributor to CV outcome in these patients. Some studies showed a higher prevalence of HT in patients with RA in comparison to sex and age-matched general population [2–4]. Furthermore, HT appears to influence CV outcome as hypertensive RA patients show an increased risk of CV events, in particular myocardial infarction, asymptomatic CV damage and left ventricular dysfunction, in comparison to normotensive patients [5].

Since BP is a highly variable biological trait [6,7], the discrepancy between different studies in terms of prevalence of HT in RA, as well as in other clinical conditions, could also reflect inherent inaccuracies in the BP measurements. For example, the alerting reaction to the medical environment, generally known as “white-coat effect” phenomenon [8–10], may lead to a variable rise in BP which can impair the precision of BP recordings. In order to overcome the “white-coat effect” phenomenon, an innovative method of BP measurement has been recently introduced, which consists of some automated office BP measurements, at about 1-minute intervals, after about 5 min of rest, with the patient left alone in the office before and during the measurements. Such method, commonly referred to as “unattended BP measurement”, has been used in the landmark *Systolic Blood Pressure Intervention Trial* (SPRINT), in which 9361 hypertensive patients were randomised to 2 different BP targets (<140 mmHg versus <120 mmHg) [11]. The trial was stopped prematurely because of the superiority of the more intensive BP target in terms of a primary composite end-point of major CV events [11]. Some data suggest that “unattended” systolic BP is about 5–10 mmHg lower than “attended” BP [12], thus complicating the application of SPRINT results to the usual clinical practice in which BP is measured by doctors or nurses in the clinic in a traditional way [13]. The “white-coat effect” phenomenon has been observed in about 30% of treated hypertensive and 20% of untreated RA patients [14]. However, the prevalence and CV consequences of “white-coat effect” phenomenon in RA population has been poorly explored.

Thus, the present study was designed to specifically compare the “unattended” with the traditional (“attended”) BP measurement technique and to investigate its association with cardiac organ damage in a cohort of RA patients.

## Materials and methods

### Study population

From February 2019 to June 2020, consecutive Caucasian RA outpatients attending the Rheumatology Unit and meeting the American College of Rheumatology (ACR) or the 2010 ACR/European League Against Rheumatism classification criteria were included in the study [15,16]. Patients not able to understand or to provide written informed consent and patients with conditions precluding a correct electrocardiography (ECG) assessment of left ventricular hypertrophy (complete right bundle branch block, left bundle branch block, atrial fibrillation, pathological Q waves due to prior myocardial infarction and Wolf-Parkinson-White syndrome) or a correct blood pressure measurement as severe obesity (body mass index ≥40) were excluded. All patients underwent a detailed medical interview and clinical examination. For the purpose of the study, the following parameters were specifically collected: anthropometric measures, smoking status (current, former, never), diabetes mellitus, history of HT and previous CV events, which included acute coronary syndrome (ST-elevation and non-ST elevation myocardial infarction and unstable angina pectoris), chronic ischaemic heart disease, cerebrovascular events (stroke or transient ischaemic attack), peripheral arterial occlusive disease with or without revascularization procedures and heart failure with reduced ejection fraction. Diabetes mellitus was defined by a fasting glucose of ≥7.0 mmol/L (126 mg/dL) or use of antidiabetic drugs. Biochemical data were retrieved from patient medical records and included erythrocyte sedimentation rate (ESR), C-reactive protein (CRP) as mean of the last three values, lipid status (cholesterol, triglycerides, high density lipoprotein (HDL)-cholesterol, low-density lipoprotein (LDL)-cholesterol), glycaemia, glomerular filtration rate and uric acid concentration. Disease-specific parameters included presence of erosions, extra-articular involvement (namely pulmonary, ocular, cutaneous, rheumatoid nodulosis and cardiac except for CV events as defined), rheumatoid factor (RF) and anti-citrullinated protein antibodies (ACPA) positivity.

The Disease Activity Score in 28-joint (DAS28)-ESR and the Clinical Disease Activity Index (CDAI) scores were assessed as measures of disease activity [17]. Patients were classified into remission, low, moderate and high disease activity according to established cut-points for these composite measures [17].

Finally, self-reported and medical chart-derived data about current use of lipid-lowering medications, anti-hypertensive drugs, insulin, insulin analogs, non-insulin oral hypoglycaemic drugs, non-steroidal anti-inflammatory drugs (NSAIDs), ongoing corticosteroids and/or conventional synthetic/biological disease modifying anti-rheumatic drugs for RA were collected.

The study was conducted in collaboration with the Cardiologic staff of the same hospital. The protocol, conforming to the ethical guidelines of the Declaration of Helsinki, was approved by the local Regional Ethical Committee (Comitato Etico Regionale-approval number 3501/19) and all patients provided written informed consent.

### BP measurements

All BP measurements were scheduled in the morning of the same day and the unattended and attended measurement techniques were performed in random order according to a cross-over design. BP was measured in the same patient both traditionally (“attended” BP) and by the “unattended” protocol. We used the same device, an Omron HEM 907 (Omron Healthcare, Lake Forrest, IL) for attended and unattended BP measurements. The device model was the same as that used in the SPRINT trial [11]. For unattended measurements, patients were left alone in a quiet room during the 5 min before the measurements and during the 3 automated measurements at distance of 1 min. The average BP was used for analysis. For attended measurements, the patients remained in a sitting position for 5 min in the presence of a doctor in the room, but without talking with them. The average BP from 3 consecutive measurements at 1-minute interval was used for analysis.

Hypertension was defined by a traditional (“attended”) BP ≥140/90 mmHg as suggested by the European Society of Cardiology/European Society of Hypertension (ESC/ESH) Guidelines [18], or ≥130/80 mmHg as suggested by the American College of Cardiology/American Heart Association (ACC/AHA) Guidelines [19], or by a current antihypertensive treatment.

### Electrocardiography

We recorded standard 12-lead ECG in all subjects during a brief end-expiratory apnoea. We defined electrographic left ventricular hypertrophy according to a validated method developed to correct for obesity [20]. The method includes an empiric correction of the Cornell voltage [21] (sum of the amplitude of the R wave in lead aVL + depth of the S wave in lead V_3_) (10) according to the following formula: Cornell-Body Mass Index (BMI) product (mV·kg/m^2^) = ((R wave amplitude in lead aVL + S wave depth in lead V_3_) × BMI) (20).

### Echocardiography

The M-mode echocardiographic study of the LV was performed under 2 D control. Only frames with optimal visualisation of interfaces and showing simultaneous visualisation of septum, LV internal diameter and posterior wall have been used for reading. We reported elsewhere the details about reading procedures and reproducibility of echocardiographic measures in our laboratory [22]. We calculated LV mass by using a necropsy validated formula and corrected it by height in metres at the power of 2.7 [23]. LVH was defined by a LV mass > 51.0 g/height(m)^2.7^) [24]. In our laboratory, the within-observer test-retest 90% interval of agreement for LV mass measurement is −16 to +14 g and the between-observer test-retest 90% interval of agreement is −20 to +18 g [25]. LV mechanics was assessed at the chamber level, as endocardial fractional shortening (FS), and the mid-wall level, according to a geometric model which takes into account the non-uniform systolic thickening of the LV wall [26]. A depressed LV function at mid-wall level is a marker of sub-clinical LV dysfunction [25,27] and a predictor of major CV events in hypertensive patients [28].

### Statistical analysis

STATA 15 (StataCorp, USA) and R software version 3 (R Foundation for Statistical Computing, Vienna, Austria. URL http://www.R-project.org) were used. Data are presented as mean ± standard deviation (SD) for continuous variables and proportions for categorical variables.

The strength of the relations between variables was assessed by partial correlation analysis [29]. Differences between two dependent correlation coefficients were evaluated according to established methods [30]. The concordance correlation coefficient was also used to compute a measurement of precision [31]. The following descriptive scale for values of the concordance correlation coefficient (for continuous variables) was employed to evaluate strength of agreement: <0.90, 0.90–0.95, and 0.95–0.99 for poor, moderate, and substantial strength of agreement, respectively [32]. Being a cross-over design experiment, we used analysis of variance (ANOVA) for a 2 × 2 crossover study to compute estimation of type of BP measurements and sequence effects [33]. In 2-tailed tests, *p* values <.05 were considered statistically significant.

## Results

The main characteristics of the 62 RA patients included in the study (mean age 67 ± 9 years, 87% female) are reported in Table 1. Mean duration of RA was 15 ± 4 years. About 40% of patients had erosive disease and in 7% of cases the disease was complicated by extra-articular involvement. In more than half of patients the disease was in remission or low activity according to DAS28 and CDAI indexes while 3% of patients were characterised by high disease activity at entry. Table 2 shows the BP, electrocardiographic and echocardiographic features of the population.

**Table 1.** Main features of the 62 RA patients included.

Variable	Value
Age (years)	67 (9)
Women, *n* (%)	54 (87)
Body mass index, kg/m^2^	27 (4.4)
Diabetes, *n* (%)	7 (11)
Current cigarette smoking, *n* (%)	14 (23)
Hypertension, *n* (%)	
ACC/AHA Definition	49 (79)
ESC/ESH Definition	41 (66)
Stroke, *n* (%)	2 (3)
Myocardial Infarction*, n* (%)	5 (8)
Congestive Heart Failure, *n* (%)	1 (2)
Arterial Occlusive Disease, *n* (%)	1 (2)
Cancer, *n* (%)	8 (13)
Chronic Obstructive Pulmonary Disease, *n* (%)	9 (15)
Chronic Renal Failure (GF*R* < 60 ml/min), *n* (%)	1 (2)
Disease duration (years)	15 (4)
Extra-articular RA, *n* (%)	4 (7)
Erosions, n (%)	24 (39)
RF positivity, *n* (%)	46 (74)
Anti-CCP positivity, *n* (%)	43 (69)
CDA*I* ≤ 2.3 (remission), *n* (%)	20 (32)
CDA*I* > 2.3 ≤ 10 (low activity), *n* (%)	26 (42)
CDA*I* > 10 ≤ 22 (moderate activity), *n* (%)	14 (23)
CDA*I* > 22 (high activity), *n* (%)	2 (3)
DAS28 < 2.6 (remission), *n* (%)	33 (53)
DAS28 ≥ 2.6 ≤ 3.2 (low activity), *n* (%)	12 (19)
DAS28 > 3.2 ≤ 5.1 (moderate activity), *n* (%)	15 (24)
DAS28 > 5.1 (high activity) (%)	2 (3)
Total Cholesterol (mg/dL)	192 (35)
HDL Cholesterol (mg/dL)	60 (13)
LDL cholesterol (mg/dL)	115 (31)
Glucose (mg/dL)	94 (19)
Uric acid (mg/dL)	4.7 (1.0)
Potassium (mEq/l)	4.4 (0.6)

RA: rheumatoid arthritis; RF: rheumatoid factor; anti-CCP: anti-cyclic citrullinated peptide antibody; HDL: high density lipoprotein; LDL: low density lipoprotein; GFR: glomerular filtration rate; CDAI: Clinical Disease Activity Index; DAS28: Disease Activity Index 28 joints.

Values are expressed as mean (±SD) otherwise indicated.

**Table 2.** Blood pressure, electrocardiographic and echocardiographic features.

Variable	Value
Office Blood Pressure (mmHg)	
Attended systolic blood pressure	130.5 (16)
Attended diastolic blood pressure	72.8 (9)
Unattended systolic blood pressure	121.7 (14)
Unattended diastolic blood pressure	68.6 (9)
Electrocardiographic features	
Height of the R wave in lead aVL (mm)	4.7 (3.3)
Depth of the S wave in lead V3 (mm)	7.2 (3.6)
Left Ventricular hypertrophy, N (%)	4 (7%)
Echocardiographic Features	
End-diastolic interventricular septum (cm)	0.96 (0.2)
Left ventricular internal diameter (cm)	5.80 (4.6)
End-diastolic posterior wall (cm)	0.85 (0.1)
Ejection Fraction (%)	71.8 (16)
Left ventricular mass (grams/height(m)^2.7^)	37.3 (9)
Left ventricular hypertrophy (%)	4 (7)

As reported in Table 3, more than half of patients were treated with methotrexate and 45% were on biologic therapies. Less than one third of patients were assuming glucocorticoid therapy at inclusion. As far as specific anti-hypertensive treatment was concerned, renin-angiotensin-system inhibitors were the most prevalent drugs, followed by beta-blockers and calcium-antagonists.

**Table 3.** Ongoing treatments of RA patients.

Variable	Value
Hydroxycloroquine, *n* (%)	7 (11)
Methotrexate, *n* (%)	35 (56)
Leflunomide, *n* (%)	6 (10)
Sulfasalazine, *n* (%)	1 (2)
Biologic therapies, *n* (%)	28 (45)
Glucocorticoids, *n* (%)	15 (24)
NSAIDs, *n* (%)	4 (6)
ACE inhibitors, *n* (%)	36 (58)
Angiotensin II receptor blockers, *n* (%)	3 (5)
Calcium-antagonists, *n* (%)	6 (10)
Diuretics, *n* (%)	5 (8)
Beta-blockers, *n* (%)	7 (11)
Statins, *n* (%)	3 (5)
Insulin, *n* (%)	4 (6)
Other hypoglycaemic drugs, *n* (%)	1 (2)

ACE: angiotensin converting enzyme; NSAIDs: non-steroidal anti-inflammatory drugs; RA: rheumatoid arthritis.

Prevalence of HT was 66% according to the ESC/ESH Guidelines, and 79% according to the ACC/AHA Guidelines. Of note, among patients with history of HTN, none had BP values < 130/80 mmHg. Furthermore, 12 patients (48%) without prior diagnosis of HTN exhibited BP values ≥ 130/80 mmHg.

Using definition of HT by ACC/AHA Guidelines, all patients classified as normotensive (or with controlled BP) by “attended” BP were also normotensive (or with controlled BP) by “unattended” BP. Conversely, among the 48 patients classified as HT (or with uncontrolled hypertension) by “attended” BP, 33 (69%) were normotensive (or with controlled BP) by “unattended” BP (white-coat effect). Main characteristics (including older age, sex, obesity, and prevalence of comorbidities) showed similar distributions between patients with or without white-coat effect (all *p* > .05).

Prevalence of diabetes was 11%. Overall, a history of myocardial infarction, congestive heart failure and arterial occlusive disease was found in 8%, 2% and 2% of patients, respectively. Finally, 13% of patients had a history of cancer and 15% a current diagnosis of chronic obstructive pulmonary disease.

Overall, 71% and 58% of patients were characterised by well controlled BP values according to the ESC/ESH Guidelines (attended BP <140/90 mmHg) and to the ACC/AHA guidelines (attended BP <130/80 mmHg), respectively.

**Figure 1.**
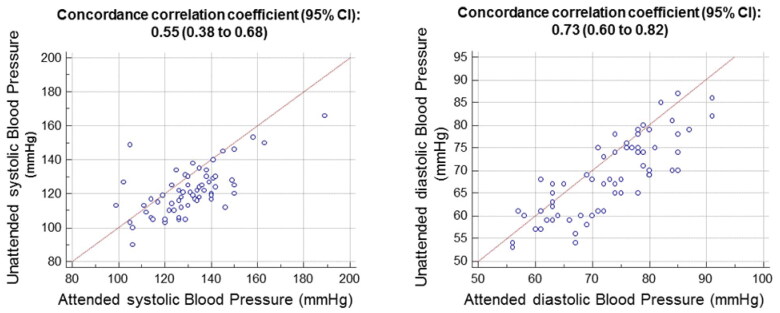
Association between unattended and attended blood pressure measurements.

**Figure 2.**
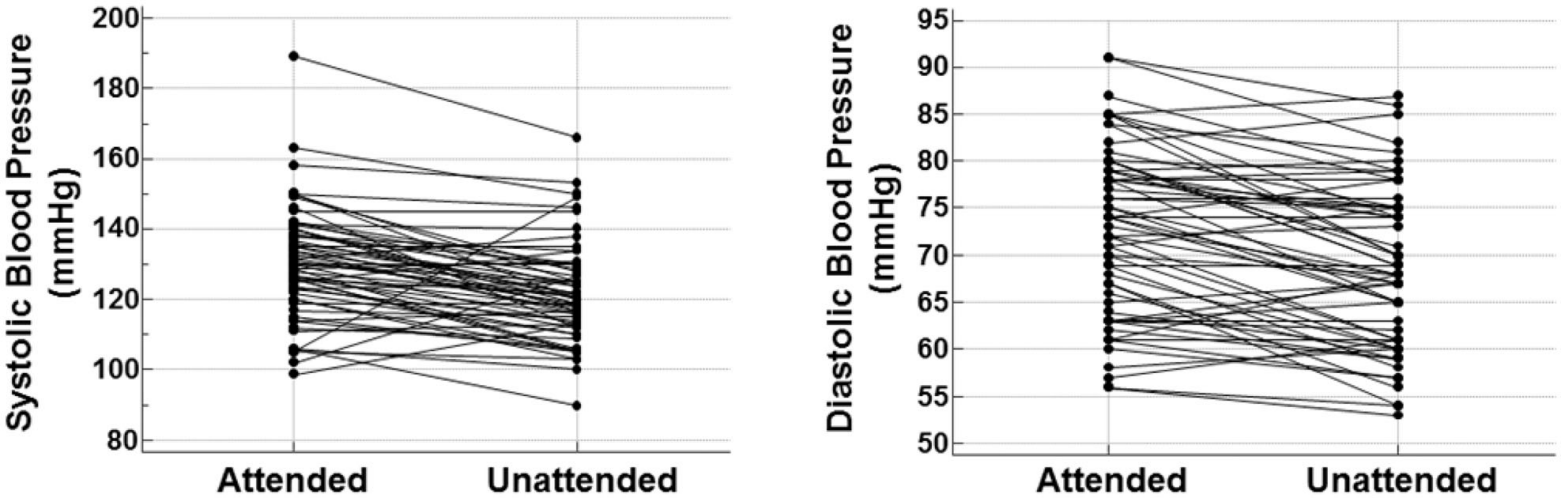
Attended and unattended blood pressure in each patient.

**Figure 3.**
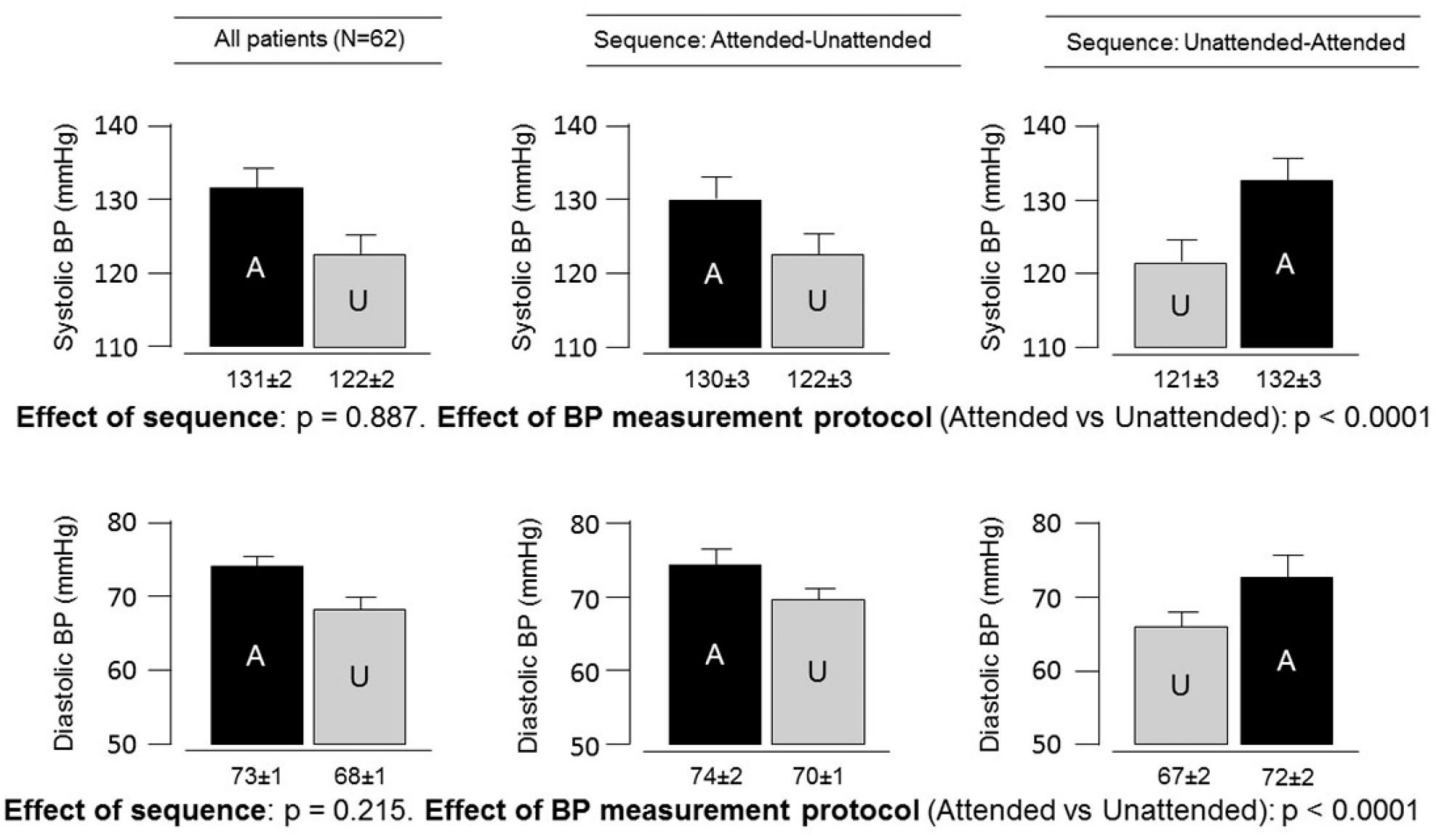
Systolic and diastolic blood pressure obtained using the traditional (attended) and unattended methods.

On average, BP was lower with the ‘unattended’ than the ‘attended’ protocol (121.7/68.6 mmHg versus 130.5/72.8 mmHg, both *p* < .0001). Figure 1 shows the concordance correlation coefficients between the two techniques, which were 0.55 (95% confidence interval 0.38–0.68) for systolic BP and 0.73 (95% confidence interval 0.60–0.82) for diastolic BP. Figure 2 shows the unattended and attended BP values in each patient. Figure 3 depicts the unattended and attended BP values in the total population and according to the two different study sequences in the cross-over design. There was no significant impact of sequence for systolic (*p* = .887) and diastolic (*p* = .215) BP. Furthermore, we tested the influence of the following covariates: age, sex, disease duration, and use of BP lowering drugs and corticosteroids. The association between “attended” and “unattended” BP (for both systolic and diastolic components) were unaffected by these variables (all *p* > .05).

**Figure 4.**
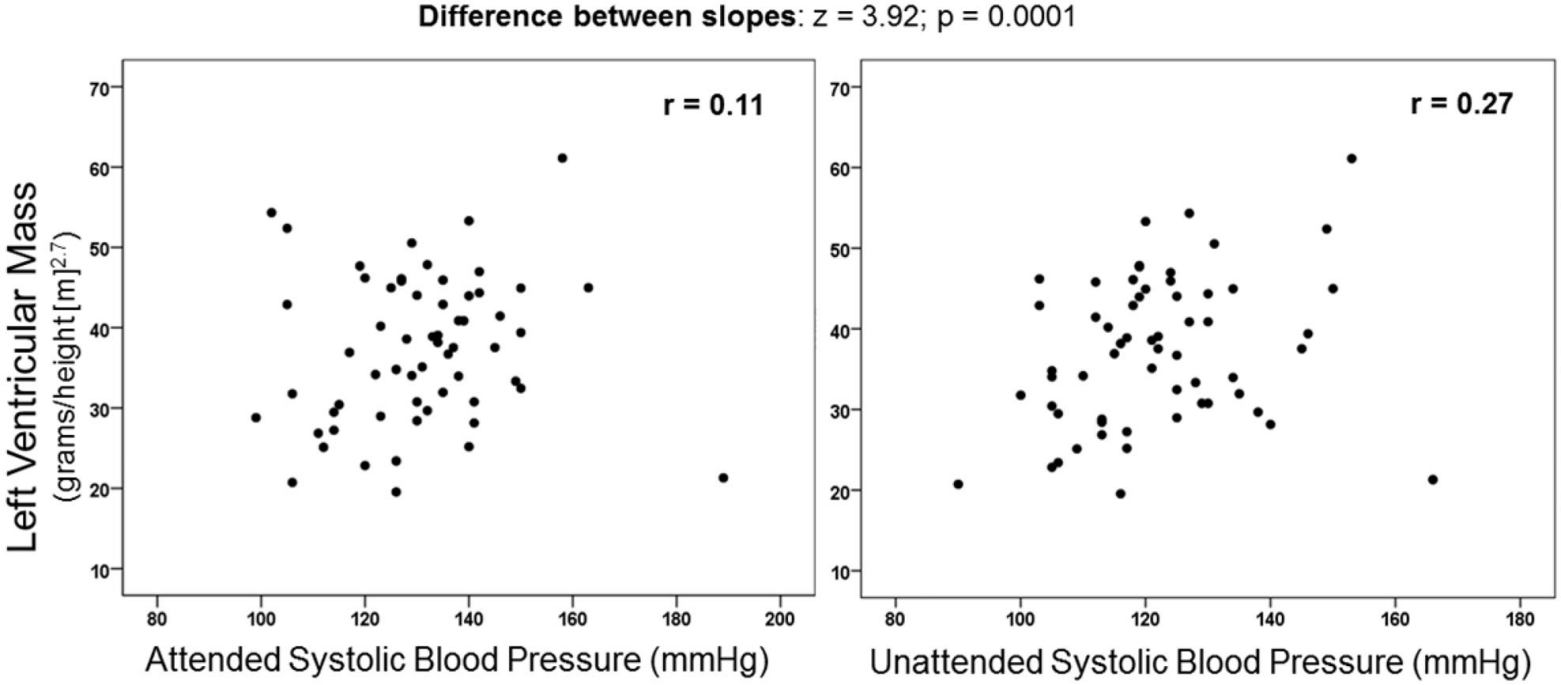
Left ventricular mass at echocardiography in relation to blood pressure obtained with traditional (attended) and unattended methods.

Figure 4 shows the association between LV mass and attended (*r* = 0.11; *p* = .40) and unattended (*r* = 0.27; *p* = .036) systolic BP. There was a generally weak association between LV mass and BP measured with the two protocols. However, the degree of association was stronger with unattended compared with attended BP (difference between slopes: *z* = 3.92; *p* = .0001). Among other characteristics of study population, only age showed a significant association with LV mass (*r* = 0.26, *p* = .044).

CDAI did not show any statistically significant association with unattended or attended systolic/diastolic BP (*r* = 0.03/*r* = 0.02 and *r* = 0.16/0.10 respectively), LV mass (*r* = 0.14) endocardial shortening fraction (*r* = 0.17) or mid-wall shortening fraction (*r* = 0.04). Similarly, DAS28 was not associated with unattended or attended systolic/diastolic BP (*r* = 0.03/*r* = 0.06 and *r* = 0.22/0.06 respectively), LV mass (*r* = 0.09) endocardial shortening fraction (*r* = 0.21) or mid-wall shortening fraction (*r* = 0.03). When disease remission was defined by CDAI ≤ 2.3 plus DAS28 < 2.6, 30% of patients were classified as in remission and the remaining 70% not in remission. There were no statistically significant differences between the two groups in terms of unattended (118/65 mmHg vs 117/65 mmHg) and attended (120/67 mmHg vs 127/71 mmHg) BP, LV mass (31 vs 35 g/height(m)^2.7^), endocardial shortening fraction (31% vs 40%) and mid-wall shortening fraction (15% vs 17%).

## Discussion

The present randomised cross-over study conducted in a cohort of RA patients shows that the “unattended” BP is about 8.8/4.2 mmHg lower than the traditional (“attended”) BP and more closely associated with LV mass, an established index of target organ damage and independent CV prognostic marker [18,34,35]. To the best of our knowledge, this is the first study which compared, in RA patients, the traditional measurement of BP with a relatively novel technique characterised by the absence of nurses or doctors in the room in the few minutes before and during the BP measurements carried out by an automated device. Strengths of our study are the random sequence of the two BP measurement techniques, the implementation of both techniques in the same day and the assessment of the structural and functional features of the LV through an echocardiographic study.

The strong epidemiological link between RA and CV disease may be related to multiple factors which include inflammatory background, immune-mediated mechanisms, concomitant therapies and the increased prevalence of traditional CV risk factors [1,36]. In this context, HT is an often underdiagnosed contributor to CV outcome in RA patients and multiple mechanisms, not only related to mechanical injury of arterial wall, may lead to an impaired BP control in these patients [5,36,37]. An association between RA and HT emerged from several studies [2,3,38], but evidence is controversial [5,37,39]. A meta-analysis of 15 case-control studies which included 2956 patients found a higher prevalence of diabetes, cigarette smoking and low HDL cholesterol levels in patients with RA, but not a higher prevalence of HT [40]. The large range of the reported HT prevalence among the different studies may be attributed to the different definitions of HT at entry and to the wide variability of populations included [5,37]. Nevertheless, HT has been recognised as a relevant contributor to adverse CV prognosis in these patients. In particular, increased LV mass and subclinical LV dysfunction, indirect signs of HT-induced organ damage, recently emerged as adverse prognostic markers in normotensive as well as hypertensive patients with RA [41–43]. Consequently, the need to improve awareness of HT among RA patients and to provide a correct and reliable method of BP measurement in this population is relevant in order to reduce long term CV morbidity. In this setting, the demonstration of the relationship between “unattended” BP values and LV mass in our cohort suggests that this novel method of BP measurement should be further investigated to support its employment in RA patients as a reliable measure of target HT-induced organ damage. Moreover, the “unattended” evaluation of BP values may provide a more accurate estimation of real prevalence of undiagnosed HT in these patients. It is well known that the clinical visit generally elicits a variable rise in BP [8], known as “white-coat effect” phenomenon, which may reflect a state of anxiety in the patient who is waiting for a “sentence” from doctors regarding his/her state of health or disease [44]. In patients with RA, a clinically important “white-coat effect” has been observed in about 30% of treated hypertensive and 20% of untreated patients [14]. Of note, regardless of concomitant anti-hypertensive treatment, the “white-coat effect” in RA patients was associated with higher prevalence of measures of subclinical vascular damage, as impaired aortic stiffness, common carotid hypertrophy and carotid plaques, thus suggesting that this phenomenon should be regarded as a risk factor for CV disease in these patients [14].

In this context, it is important to investigate which type of BP measurement is more closely associated with target organ damage and, hopefully, prognosis. In addition to 24-h BP monitoring [45] and self-measured BP at home [46], the technique of “unattended” BP measurements has been recently introduced, and implemented in the SPRINT study [11]. Our study confirms the suggestion by Kjldsen and Mancia that the “unattended” systolic BP is 5–10 mmHg lower than attended BP [13] and extends this finding to patients with RA. In a study by Salvetti et al., in which “unattended” and “attended” BP values were measured in the same day according to a cross-over design in mixed population of hypertensive and normotensive subjects, “unattended” BP was about 6/2 mmHg lower than “attended” BP [47]. At variance with Salvetti and co-workers, in our study the LV mass showed a slightly closer association with “unattended” than with “attended” systolic BP, although the closeness of association was rather weak with both techniques of measurements. The discrepancy between the two studies might be accounted for by the slight difference between “unattended” and “attended” BP in the two studies (130.5 vs 121.7 mmHg in our study versus 134.5 vs 128.0 mmHg in the study by Salvetti and coworkers [47]) and by the single-center design in our study. Similar degrees of association between LV mass and “unattended” versus “attended” BP have been noted in other two studies which, however, did not measure BP with the two techniques during the same day, thereby raising the possibility of a systematic bias [48,49].

In our study, we did not detect any association between disease activity and “attended” or “unattended” BP values. Disease activity, moreover, was not associated with echocardiographic measures of organ damage, at first glance contrasting previous reports in RA patients [50]. Left ventricular mass is an estimation of the ventricular weight and is thought to represent the cumulative effect of BP on the heart. The different measures of LV strains employed in the study by Midtbø H *et al* as compared to the present study may account for such inconsistency and reflect possible different effects of inflammatory cytokines on myocardial structure. Moreover, the cross-sectional nature of the study and the punctual evaluation of disease activity preclude from quantifying the burden of inflammation patients were exposed over time and its long term effect on myocardial structure. Finally, other limitations include the small sample size of our cohort and the evidence that majority of our patients were in remission to low disease activity at inclusion. In this setting, a recent study evaluating cardiac resonance imaging indices of structure, function and fibrosis in a cohort of RA patients with low to moderate disease activity did not found evidence of increased diffuse myocardial fibrosis or inflammation [51], reinforcing a recent demonstration that traditional CV risk factors, in particular HT, rather than RA-specific features appear to be stronger determinants of impaired systolic function in these patients [52]. Notably, our cohort included long-standing patients in good disease control with near to half on treatment with biological therapies. Thus, we cannot exclude an effect of biological therapies on ventricular mass and function through the reduction of disease activity and systemic inflammation [53]. Moreover, we did not quantify the cumulative exposure to corticosteroid therapy, a variable known to be associated with increased BP values and higher LV mass. Nevertheless, less than one third of our patients were assuming corticosteroid therapy at inclusion and at low dose (≤7.5 mg/day of prednisone or equivalent), thus not excluding a potential paradoxical cardioprotective effect of low-dose corticosteroids through their anti-inflammatory activity on the vessel wall [54]. Similarly, the results could be biased by some pharmacologic therapy, including non-steroidal anti-inflammatory drugs (NSAIDs), which may have negative effect on BP values and CV outcome measures. However, only 6% of patients were assuming NSAIDs at study inclusion and use of this class of drugs is usually intermittent or sporadic, thus challenging the real assessment of NSAID effects.

In conclusion, the present study highlights the importance of a correct measurement of BP in patients with RA and provides the first demonstration that, by reducing or eliminating the “white-coat effect” phenomenon, the “unattended” BP measurement may be a reliable method for evaluation of BP values in these patients as it strictly correlates with measures of HT-induced target organ damage. Given the relevance of HT as CV risk factor in RA patients and its detrimental role on CV outcome, we suggest that this method may be employed in future studies aimed to assess prevalence of HT in RA patients. Larger prospective studies are needed to evaluate accuracy and reproducibility of the novel “unattended” BP measurement in comparison with the traditional measurement in patients with systemic rheumatic diseases.

## Data Availability

The data that support the results of the current study are available upon reasonable request and are accessible from the corresponding Author.
